# Anti-angiogenic and anti-tumor effects of metronomic use of novel liposomal zoledronic acid depletes tumor-associated macrophages in triple negative breast cancer

**DOI:** 10.18632/oncotarget.20539

**Published:** 2017-08-24

**Authors:** Xin-Jun Cai, Zeng Wang, Jia-Wei Cao, Jian-Jun Ni, Ying-Ying Xu, Jun Yao, Hong Xu, Fang Liu, Gao-Yi Yang

**Affiliations:** ^1^ Department of Pharmacy, Zhe Jiang Chinese Medicine and Western Medicine Integrated Hospital, Hangzhou 310003, People's Republic of China; ^2^ Department of Pharmacy, Zhejiang Cancer Hospital, Hangzhou 310022, People's Republic of China; ^3^ Department of Gastroenterology and Hepatology, Integrated Chinese and Western Medicine Hospital of Zhejiang Province, Hangzhou 310003, People's Republic of China; ^4^ Department of Acupuncture, Integrated Chinese and Western Medicine Hospital of Zhejiang Province, Hangzhou 310003, People's Republic of China; ^5^ Department of Ultrasound, Integrated Chinese and Western Medicine Hospital of Zhejiang Province, Hangzhou 310003, People's Republic of China

**Keywords:** zoledronic acid, tumor-associated macrophages, triple negative breast cancer, anti-angiogenic, anti-tumor effects

## Abstract

Zoledronic acid (ZOL) has been used as an adjuvant therapy for breast cancer. It is suggested that ZOL might be associated with inhibition of macrophages, which in turn reduces tumor growth, metastasis and tumor angiogenesis. Moreover, metronomic therapy can inhibit tumor angiogenesis and tumor immune cells. Previously we developed ZOL based cationic liposomes that allowed a higher intratumor delivery of drug compared with free ZOL *in vivo*. Therefore, in this study, Asn-Gly-Arg (NGR) and PEG2000 were used as ligands to modify the surface of liposomes (NGR-PEG-LP-ZOL) in metronomic therapy to clear the tumor-associated macrophages (TAMs) and inhibit the formation of tumor angiogenesis, achieving the purpose of anti-tumor growth.

Our data showed that NGR-PEG-LP-ZOL metronomic therapy has the strongest inhibitory effect on tumor growth. Further, NGR-PEG-LP-ZOL metronomic therapy could significantly impair TAMs by inhibiting the expression of CD206 antibody in tumor tissues, decreasing the expression of cytokine related gene expression of TAMs, as well as reducing the percentage of TAMs in tumor tissues. In addition, NGR-PEG-LP-ZOL metronomic therapy could significantly inhibit the expression of tumor neovascular specific antibody CD31 and reduce the microvessel density. In conclusion, our study demonstrated that NGR-PEG-LP-ZOL metronomic therapy could impair TAMs by inhibiting tumor angiogenesis and enhance the antitumor effect of ZOL.

## INTRODUCTION

Breast cancer is one of the leading causes of cancer death among women [1]. Based on the treatment scheme, breast cancer is divided into three types, (1) HER2-positive; (2) hormone receptor-positive (ER^+^ and/or PR^+^), (i.e. luminal A and B); and (3) triple-negative (ER^-^, PR^-^, HER2^-^)[2]. Among these breast cancer patients, 15–20% of patients are characterized as triple-negative breast cancer (TNBC) phenotype, namely, the absence of estrogen receptors, progesterone receptors and human epidermal growth factor receptor 2 [3]. Compared to breast tumors of other molecular subtypes, TNBC is more aggressive and have a poor prognosis [3]. The triple-negative nature renders TNBC patients non-respondent to therapies that target HER2 receptors or to hormonal therapies. The only systemic therapy for patients with TNBC is adjuvant chemotherapy, which include various combinations of anthracyclines, taxanes, or cyclophosphamide [4]. However, further studies revealed that the treatment effect of existing chemotherapy regimens is still unsatisfactory [5].

Herein we report a potential targeted therapy for TNBC by targeting tumor-associated-macrophages (TAMs). TAMs are a kind of stromal cells, which involves major population of tumor macrophages, and recruit into the tumor microenvironment by a series of soluble cytokines and chemokines such as macrophage colony stimulating factors [6, 7]. Moreover, CDl63, CD204 and CD206 are the biomarkers present on the TAMs [8, 9]. However, TAMs secrete characteristic phenotypic molecules, such as Arg1, Fizz1, Msr2, Fra1, Ym1, CCL3, CCL22 and so on [10, 11, 12, 13]. Recently, strong clinical evidence suggest that TAMs are not only involved in tumor invasion, growth, angiogenesis, metastasis, immune suppression, but can also stimulate the formation of new blood vessels, degradation of matrix, local invasion and distant metastasis [14, 15]. More importantly, TAMs in TNBC are associated with higher risk of tumor progression and distant metastasis. Yuan et al found that TNBC with large number of infiltrating TAMs demonstrated significantly higher risk of distant metastasis, as well as lower rates of disease-free survival and overall survival than those with smaller number of infiltrating TAMs [16]. Therefore, modifying the activity and/or number of TAMs is considered as a viable target for cancer therapy.

Bisphosphonates such as zoledronic acid (ZOL) has been frequently used for treating bone diseases in cancer patients with bone metastasis [17]. In addition to the anti-resorptive efficacy, ZOL was used in patients with solid tumors, such as breast cancer, lung cancer, prostate cancer which could increase the antitumor effects [18, 19, 20]. Moreover, ZOL could inhibit the ability of TAMs to recruit and impair the number of TAMs, thereby reducing the burden of metastases [21].

A new method to strengthen the anti-tumor effects of ZOL is by administering the drug in a metronomic way with frequent administration of low doses of drugs and with shorter intervals of consecutive doses [22]. Metronomic use of low dose chemotherapeutic drugs could inhibit angiogenesis, reduce the level of microvessel density (MVD) and the expression of tumor angiogenesis specific proteins, VEGF and CD31 [23]. Recently, preclinical and clinical studies regarding the metronomic use of low dose ZOL demonstrated more anti-tumor efficacy than conventional therapy in breast cancer patients in the reduction of biomarkers, such as NTx and VEGF [24]. In addition, ZOL is rapidly cleared by the kidney, while 50% of the administered dose was retained in the bone mineral matrix where the osteoclast-inhibitory effect takes place after intravenous administration [25]. To address this problem, we prepared ZOL cationic liposomes that allowed a higher intratumor delivery of the drug compared with free ZOL *in vitro* and *in vivo* [26]. Nevertheless, there were very few reports present on the effect of metronomic ZOL liposomes in breast cancer. Therefore, in this study, we upgraded the previously developed liposomes by introducing PEG2000 and one peptide containing Asn-Gly-Arg (NGR) as ligands on the surface of liposomes. NGR could recognize a specific isoform of aminopeptidase N (APN), which is a membrane-bound, zinc-dependent metalloproteinase that plays a key role in tumor invasion and angiogenesis, and has been identified as a potent targeting ligand for the delivery of chemotherapeutic drugs [27]. Therefore, we investigated the possibility of depletion of TAMs and anti-angiogenicity by ZOL entrapped in the NGR-modified PEG2000-liposomes could increase antitumor effects when administered as metronomic therapy compared to conventional therapy.

## RESULTS

### Characterization of liposomes

The average particle size of NGR-PEG-LP-ZOL was approximately (105±12) nm, the polydispersity was (0.15±0.06). The zeta potential value of NGR-PEG-LP-ZOL was close to neutrality (-1.89±0.21 mV). The encapsulation efficiency of NGR-PEG-LP-ZOL was (10.42±0.23)%, and the drug loading percentage of NGR-PEG-LP-ZOL was (2.69±0.35)%

**Figure 1:**
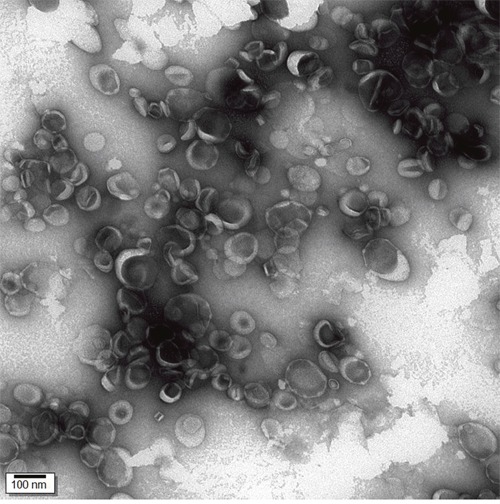
Transmission electron photomicrographs of NGR-PEG-LP-ZOL Particles were imaged using an accelerating voltage of 75 kV (magnifcation: ×150 000). The morphological characteristics of NGR-PEG-LP-ZOL were generally spherical and regular in size.

As shown in Figure 1, the morphological characteristics of NGR-PEG-LP-ZOL group were generally spherical and regular in size.

### *In vivo* anti-tumor activity

**Figure 2:**
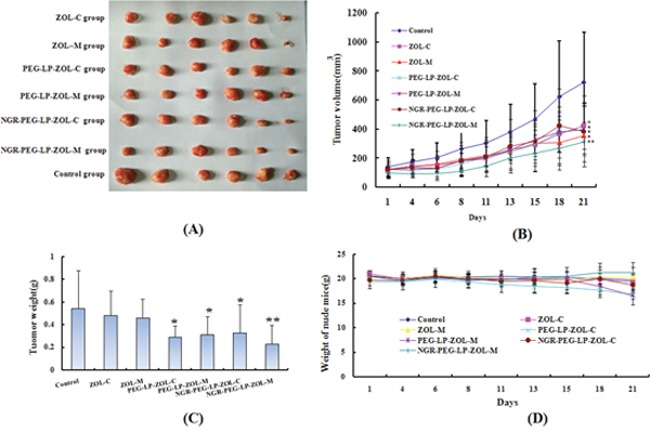
Effect on anti-tumor activity *in vivo* Six weeks-old female BALB/c mice bearing 300 mm^3^ MDA-MB-231 tumors were randomly divided into the following groups (6 mice/group): (1) Control group (physiological saline, i.v.), (2) ZOL-C (0.1 mg/kg, i.v., q7d), (3) ZOL–M (ZOL equivalent dose of 0.025 mg/kg, i.v., q2d), (4) PEG-LP-ZOL-C (ZOL equivalent dose of 0.1 mg/kg, i.v., q7d), (5) PEG-LP-ZOL-M (ZOL equivalent dose of 0.025 mg/kg, i.v., q2d), (6) NGR-PEG-LP-ZOL-C (ZOL equivalent dose of 0.1 mg/kg, i.v., q7d), (7) NGR-PEG-LP-ZOL-M (ZOL equivalent dose of 0.025 mg/kg, i.v., q2d). The total dose of ZOL in all therapy groups was 0.4 mg/kg. All drugs were administered after dissolving in water for injection. **(A)** Photographs of representative tumors from each treatment group after mice was sacrificed. **(B)** Tumor growth was monitored three times a week by caliper measurement. Results were presented as mean ± standard deviation (SD), (n =6). (^**^p < 0.01 vs control group; ^*^p < 0.05 vs control group). **(C)** Examination of tumor weight at the time of sacrifice and 21 days post-inoculation. Results were presented as mean ± standard deviation (SD), (n =6). (^**^p < 0.01 vs control group; ^*^p < 0.05 vs control group). **(D)** Graph of nude mice weight change in the experimental period.

The anti-tumor effect of ZOL formulations was evaluated in MDA-MB-231 breast tumor-bearing mice after cell implantation. As shown in Figure 2A, xenografts derived from NGR-PEG-LP-ZOL metronomic therapy (NGR-PEG-LP-ZOL-M) group demonstrated slow growth compared to other groups. As shown in Figure 2B, the tumor growth was inhibited in all therapy groups compared with the control group, but the effect obtained varied. Compared with the control group, NGR-PEG-LP-ZOL-M demonstrated strongest inhibitory effect on tumor growth (p<0.01).

The ZOL conventional therapy (ZOL-C) inhibited tumor growth, but showed no statistically significant difference compared with control group (p>0.05). In addition, ZOL metronomic therapy (ZOL-M), PEG-LP-ZOL conventional therapy (PEG-LP-ZOL-C), PEG-LP-ZOL metronomic therapy (PEG-LP-ZOL-M), NGR-PEG-LP-ZOL conventional therapy (NGR-PEG-LP-ZOL-C) inhibited tumor growth significantly compared with control group (p<0.05). However, NGR-PEG-LP-ZOL-M showed no stronger anti-tumor effect than other therapy groups (p>0.05).

The average tumor weight in the control group, ZOL-C, ZOL-M, PEG-LP-ZOL-C, PEG-LP-ZOL-M, NGR-PEG-LP-ZOL-C, and NGR-PEG-LP-ZOL-M on day 28 was (0.54±0.34), (0.31±0.21), (0.36±0.16), (0.29±0.09), (0.31±0.16), (0.32±0.24), (0.23±0.16) g, respectively (Figure 2C). The values of TWI (%) in all therapy groups compared with control group were displayed in Table 1. In addition, no significant weight loss was observed between the therapy groups and the control group (Figure 2D).

**Table 1:** The value of TWI (%) in all therapy groups

Treatment groups	TWI (%)
control	/
ZOL-C	42.45
ZOL-M	33.37
PEG-LP-ZOL-C	46.32
PEG-LP-ZOL-M	42.59
NGR-PEG-LP-ZOL-C	39.4
NGR-PEG-LP-ZOL-M	57.4

### Effect on the density/population of TAMs *in vivo*

**Figure 3:**
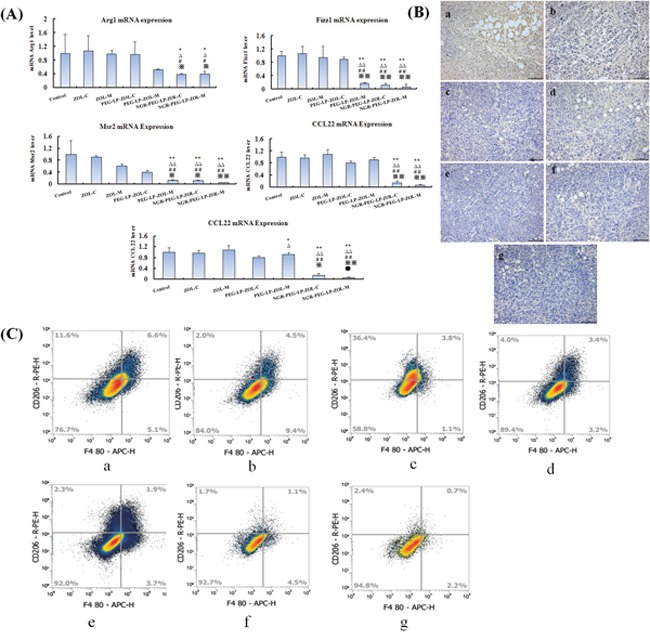
Effect on the density/population of TAMs *in vivo* **(A)** The expression levels of Arg1, Fizz1, Msr2, CCL3, CCL22 mRNA, which were markers of TAMs in tumors. The mRNA was analyzed by RT-qPCR. Results were presented as mean ± standard deviation (SD) (n =6). (^**^p< 0.01 vs control group; ^*^p< 0.05 vs control group;^ΔΔ^p < 0.01 vs ZOL-C group; ^Δ^p< 0.05 vs ZOL-C group;^##^p< 0.01 vs ZOL-M group; ^#^p < 0.05 vs ZOL-M group; ^※※^p<0.01 vs PEG-LP-ZOL-C group; ^※^p<0.05 vs PEG-LP-ZOL-C group; ^●●^p<0.01 vs PEG-LP-ZOL-M group; ^●^p<0.05 vs PEG-LP-ZOL-M group). **(B)** Tumor sections from each group of animals were immunostained for CD206, a marker of TAMs. The photographs are representative of sections from 6 tumors/group (×100). Note: a:Control group; b:ZOL-C; c:ZOL–M; d:PEG-LP-ZOL-C; e:PEG-LP-ZOL-M; f:NGR-PEG-LP-ZOL-C; g:NGR-PEG-LP-ZOL-M **(C)**. Percentage of TAMs in MDA-MB-231 tumors in mice treated with PBS, ZOL-C, ZOL–M, PEG-LP-ZOL-C, PEG-LP-ZOL-M, NGR-PEG-LP-ZOL-C, NGR-PEG-LP-ZOL-M. Mice that were treated with PBS were used as controls. Tumor cell suspensions were stained with APC-labeled anti-CD206 and were analyzed by using a flow cytometer. Note: a:Control group; b:ZOL-C; c:ZOL–M; d:PEG-LP-ZOL-C; e:PEG-LP-ZOL-M; f:NGR-PEG-LP-ZOL-C; g:NGR-PEG-LP-ZOL-M.

As the quantity and activity of TAMs could be explained by the expression of Arg1, Fizz1, Msr2, CCL3, CCL22, and so the mRNA levels of these factors in tumor tissues were detected [10, 11, 12, 13]. Therefore, we investigated the expression of Arg1, Fizz, Msr2, CCL3, CCL22 mRNA in tumor cells using RT-qPCR. As shown in Figure 3A, ZOL-C or ZOL-M or PEG-LP-ZOL-C showed no effect on inhibition of the expression of Arg1, Fizz1, Msr2, CCL3, CCL22. Moreover, whether conventional therapy or metronomic therapy, NGR-PEG-LP-ZOL therapy group was obviously more effective than the ZOL or PEG-LP-ZOL by significantly inhibiting the expression of Arg1, Fizz, Msr2, CCL3, CCL22, respectively (p<0.05 or p<0.01).

To assess the affect of different formulations of ZOL therapy on the density/population of TAMs, we used CD206 immunostaining to evaluate the expression of TAMs at the tumor site. According to Figure 3B, we found that a less number of CD206 positive macrophages were stained in the metronomic therapy groups (ZOL or PEG-LP-ZOL) compared with the conventional therapy groups. For metronomic therapy, NGR-PEG-LP-ZOL therapy group showed a significant reduction in CD206^+^ TAMs expression compared to ZOL. For conventional therapy, NGR-PEG-LP-ZOL therapy showed a slight reduction in CD206^+^ TAMs expression compared to ZOL or PEG-LP-ZOL therapy.

As shown in Figure 3C and in Table 2, NGR-PEG-LP-ZOL-M could significantly reduce the percentage of TAMs. For metronomic therapy, the NGR-PEG-LP-ZOL therapy was significantly more effective than the ZOL therapy in reducing CD206^+^ TAMs. For conventional therapy, the NGR-PEG-LP-ZOL and PEG-LP-ZOL therapy groups were significantly more effective than the free ZOL therapy in reducing the percentage of CD206^+^ TAMs (p<0.05 or p < 0.01). In conventional therapy, the NGR-PEG-LP-ZOL therapy was significantly more effective than the ZOL or PEG-LP-ZOL therapy groups in reducing the percentage of TAMs (p<0.05 or p<0.01). Moreover, PEG-LP-ZOL-M also reduced the percentage of CD206^+^ TAMs, but to a lesser extent than the NGR-PEG-LP-ZOL-M.

**Table 2:** The percentage of TAMs in tumors

Treatment groups	TAMs(%)
control	6.6±1.2
ZOL-C	4.5±0.6
ZOL-M	3.8±0.9
PEG-LP-ZOL-C	3.4±0.6
PEG-LP-ZOL-M	1.9±0.7^abd^
NGR-PEG-LP-ZOL-C	1.1±0.3^abde^
NGR-PEG-LP-ZOL-M	0.7±0.3^abce^

Note: (^a^p < 0.01 vs control group, ^b^p < 0.01 vs ZOL-C group, ^c^p < 0.01 vs ZOL-M group, ^d^p < 0.05 vs ZOL-M group, ^e^p < 0.05 vs PEG-LP-ZOL-C group).

Each data represents the mean±standard deviation (n=6).

### Effect on the angiogenesis *in vivo*

To evaluate the anti-angiogenic activity of different formulations of ZOL therapy *in vivo*, the MVD was assessed by immunohistochemistry using CD31 marker.

**Figure 4:**
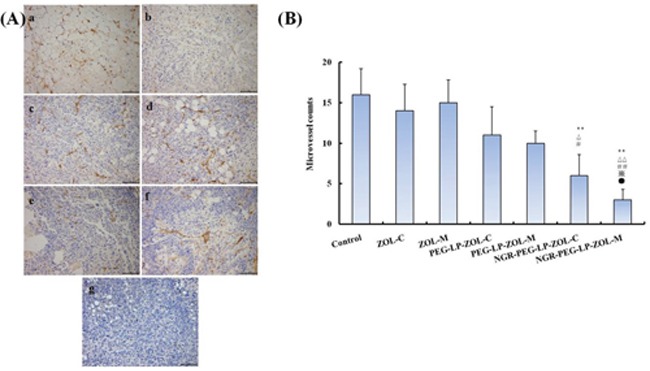
Effect on the angiogenesis *in vivo* **(A)** Effect of NGR-PEG-LP-ZOL-M on microvessel density (MVD) in xenograft MDA-MB-231 tumors. Representative micrographs of immunohistochemical detection of CD31-positive microvessels in xenograft MDA-MB-231 tumors from different therapy groups. Results were presented as mean ± standard deviation (SD) (n =6). (^**^p < 0.01 vs control group; ^*^p < 0.05 vs control group; ^ΔΔ^p < 0.01 vs ZOL-C group; ^Δ^p < 0.05 vs ZOL-C group;^##^p < 0.01 vs ZOL-M group; ^#^p < 0.05 vs ZOL-M group; ^※^p<0.05 vs PEG-LP-ZOL-C group; ^●^p<0.05vs PEG-LP-ZOL-M group). Note: a: Control group; b:ZOL-C; c:ZOL–M; d:PEG-LP-ZOL-C; e:PEG-LP-ZOL-M; f:NGR-PEG-LP-ZOL-C; g:NGR-PEG-LP-ZOL-M. **(B)** Tumor sections from each animal group were immunostained for CD31, a marker of tumor microvessel. The photographs are representative of sections of 6 tumors/group (×100).

As shown in Figure 4A. very few microvessels were observed in the metronomic NGR-PEG-LP-ZOL therapy group. There was significantly less MVD in the metronomic therapy groups (ZOL or PEG-LP-ZOL) compared with the conventional therapy groups (p<0.05). For metronomic therapy, the NGR-PEG-LP-ZOL therapy group was significantly more effective than the ZOL or PEG-LP-ZOL therapy groups in inhibiting MVD (p < 0.05). In conventional therapy groups, the NGR-PEG-LP-ZOL therapy group was slightly more effective than the ZOL or PEG-LP-ZOL therapy in inhibiting MVD, but the differences failed to reach statistical significance (p>0.05).

## DISCUSSION

According to the pre-clinical as well as clinical trials, ZOL demonstrated anti-tumor activity in different types of tumors [18, 21]. However, the authors mainly explained these anti-tumor effects by direct toxicity on the tumor cells and to a lesser extent on the tumor microenvironment in the primary tumor. These clinical trials also demonstrated that ZOL therapy increased not only tumor-free but also overall survival [19]. Rietkötter et al reported that ZOL could diminish the amount of TAMs in the primary tumor and vascularization [28]. Nevertheless, ZOL was easily absorbed by the bone and an insufficient intratumor concentrations were reached [25]. To overcome this criticism, self-assembling of nanoparticles or PEG-liposomes or cationic liposomes were used to successfully deliver ZOL in different tumor cells, with a stronger anti-cancer effect [26, 29, 30]. These anti-cancer effects were confirmed *in vivo* in an experimental model of cancer. On the basis of these results, we prepared NGR-PEG-LP-ZOL with the aim to evaluate its potential in targeting APN receptors expressed in tumor endothelial cells [31]. Our current *in vivo* results of antitumor effects demonstrated that NGR-PEG-LP could significantly improve the inhibition of breast tumor (p<0.01), compared to the control group.

Over the last decade there has been an increasing interest in the use of so-called low dose metronomic drug administration compared with conventional therapy [12]. According to the previous clinical studies, metronomic therapy has achieved good results in terms of both primary systemic therapy and maintenance therapy in a variety of cancers, such as breast cancer, prostate cancer, melanoma, pancreatic cancer, and so on [32]. The effectiveness of metronomic regimen in patients with different tumor types included prolonged survival, improved quality of life, and reduced adverse reactions. Accumulating evidence suggests that metronomic therapy suppressed tumor growth by influencing innate and adaptive immune responses [33, 34]. Metronomic therapy can reduce immune suppressive populations of CD4^+^CD25^+^ regulatory T cells (Tregs) [35]. Moreover, metronomic administration could also have effects on other subsets of immune cells. For example, Doloff et al reported that cyclophosphamide metronomic therapy could activate innate immune cells, such as natural killer (NK) cells, dendritic cells and macrophages. Comito et al found that metronomic therapy could inhibit TAMs activity by reducing metastasis and invasion of tumor, suggesting that TAMs induced immunosuppression is an important cause of tumor metastasis [36]. Several studies have reported effects of ZOL on TAMs in different tumor types *in vivo* [28, 37]. Coscia and colleagues reported that 100 μg/kg ZOL administered for 4 weeks followed by 3 weeks rest (average of 16 injections) increased both tumor free as well as overall survival and demonstrated a significant reduction in tumor growth rate and multiplicity of mammary tumors compared to control in BALB-neuT mouse mammary carcinoma model [38]. Taken together, these results suggest that ZOL metronomic administration may be helpful in improving the inhibition of TAMs. However, our results showed that free ZOL metronomic administration and conventional administration could suppress tumor growth, but showed no effect on TAMs. When compared with conventional administration, NGR-PEG-LP-ZOL and PEG-LP-ZOL metronomic administration showed no significant reduction in the expression of CD206 specific protein in tumor tissues as well as the mRNA expression of TAMs phenotypic molecules (Arg1, Fizz, Msr2, CCL3, CCL22), but reduced the percentage of TAMs. The above results showed that free ZOL demonstrated that the bone can easily absorb, but unable to reach the tumor tissues, could not play a role on TAMs. Our results were consistent with previous studies. NGR-PEG-LP-ZOL or PEG-LP-ZOL are conducive to the delivery of ZOL to the tumor tissues, and play a role on TAMs. Our data demonstrated that the metronomic NGR-PEG-LP-ZOL group reduced TAMs more markedly compared with the metronomic PEG-LP-ZOL therapy groups, suggesting better target effects of NGR-PEG-LP-ZOL in the delivery of drugs to the tumor tissues.

Anti-tumor angiogenesis is a new treatment strategy for tumor therapy, both preclinical and clinical studies have shown that inhibition of tumor angiogenesis can inhibit tumor growth. Klement confirmed that sustained and low dose of vincristine inhibited tumor angiogenesis and reduced tumor size [39]. Several researchers found that by reducing the quantity and activity of circulating endothelial progenitor cells (circulating endothelial progenitor, CEPs), metronomic administration inhibited the tumor vascular endothelial cells formation and tumor angiogenesis *in vitro* [40]. Immunohistochemistry results confirmed the anti-angiogenic effect of metronomic administration of NGR-PEG-LP-ZOL and PEG-LP-ZOL *in vivo*. We also observed anti-angiogenic effects in the NGR-PEG-LP-ZOL or PEG-LP-ZOL therapy groups, but the effect was much lower than that in the metronomic therapy group (p<0.01) as shown by the MVD evaluation.

According to previous studies, the progression of TNBC was closely related to TAMs and tumor angiogenesis. TAMs may promote the development of TNBC by promoting tumor angiogenesis [14]. Studies have shown that inhibition of TAMs maturation and invasion of tumors can delay angiogenesis and tumor progression, providing evidences for the causal relationship between tumor angiogenesis and TAMs [15, 16]. Our study found the role of ZOL in the inhibition of TAMs and decrease of MVD, which were better compared to other groups. But the mechanism of NGR-PEG-LP-ZOL metronomic administration in the inhibition of TAMs tumor angiogenesis need to be further explored.

In conclusion, our data showed that metronomic regimen of NGR-PEG-LP-ZOL (i.e. total administered dose remains the same as the clinically relevant dose) has stronger tumor inhibition effect. Further analysis of its mechanism showed that: 1) metronomic regimen of NGR-PEG-LP-ZOL can reduce the quantity and expression of TAMs in tumor microenvironment, inhibiting TAMs induced tumor growth, invasion and metastasis; and 2) it can inhibit the proliferation of tumor angiogenesis and suppress the tumor growth. However, TAMs may promote tumor angiogenesis and are closely associated with cancer progression.

Above all, we believe that NGR-PEG-LP-ZOL metronomic administration can be used as a complementary technology to improve the effectiveness of TNBC chemotherapy. But the exact underlying mechanism of NGR-PEG-LP-ZOL on TAMs needs further research.

## MATERIALS AND METHODS

### Reagents

ZOL was kindly provided by CTTQ Medicine Co., Ltd. (Jiangsu, China). Soyabean phosphatidylcholine (SPC) was purchased from Lipoid GmbH (Ludwigshafen, Germany). 1, 2-Distearoyl-sn-glycero-3-phosphoethanolamine [methoxy(polyethylene glycol)-2000] (mPEG-DSPE) was provided by Fangshuo Co., Ltd. (Shanghai, China). The peptide containing Asn-Gly-Arg (NGR) was provided by ChinaPeptides Co., Ltd. NGR-PEG-DSPE was synthesized in ChinaPeptides Co., Ltd. All other analytical grade chemicals were purchased from Huadong Pharmaceutical Co., Ltd. (Hangzhou, China).

### Cells and animal model

The human breast cancer cell line MDA-MB-231 was purchased from the Type Culture Collection of the Chinese Academy of Sciences, Shanghai, China. Four to six weeks old female BALB/c nude (nu/nu) mice weighting 20-22 g were purchased from Zhejiang University Animal Laboratory (Hangzhou, China). All procedures involving animals and their care were conducted in accordance with the institutional and governmental guidelines. The animals were housed in cages and had free access to tap water and standard laboratory food throughout the experiments.

### Preparation of NGR-modified liposomes containing ZOL (NGR-PEG-LP-ZOL)

The NGR-modified liposomes containing ZOL (NGR-PEG-LP-ZOL) were prepared by thin-film hydration method as described previously [26]. Briefly, the mixture of cholesterol, SPC, mPEG-DSPE and NGR-PEG-DSPE were dissolved in chloroform, and the resulting solution was added to a round-bottomed flask. The solvent was then removed under reduced pressure by a rotary evaporator (R202, Shanghai shensheng Instrument Co., Ltd.). The lipid film was hydrated with buffer phosphate (pH=7.6) containing ZOL and the resulting suspension was gently mixed in the presence of glass beads until the lipid layer was removed from the glass wall, the liposome suspensions were then sonicated and extruded thrice through a 0.22μm polycarbonate membrane to reduce the vesicle size.

### Characterization of NGR-PEG-LP-ZOL

The mean diameter of NGR-PEG-LP-ZOL was determined by photon correlation spectroscopy (Malvern Instruments Ltd., Worcestershire, UK). The zeta potential (ζ) of the liposome surface was measured in water by means of a Zetasizer Nano Z (Malvern, UK). The morphological examination of NGR-PEG-LP-ZOL was performed using transmission electron microscopy (TEM, JEM1200, Japan) using 2% phosphotungstic acid solution negative staining.

Encapsulation efficiency (EE%) was determined according to a previously reported method [26] (18). Briefly, 1 ml of liposome dispersions was eluted with PBS (pH 7.4) through Sephadex G-100 column to remove the unloaded ZOL. The entrapped drug was determined by disrupting the liposome dispersions with ethanol (the ratio of volume of methanol to liposome was 5:1). Drug in liposomes was measured by using high-performance liquid chromatography (HPLC). The encapsulation efficiency of ZOL was estimated as,

$$EE\%=\left(\frac{W_{columns}}{W_{total}}\right)\times 100\%$$

The drug loading (DL%) percentage of ZOL was estimated as,

$$DL\%=\left(\frac{W_{columns}}{W_{NGR-PEG-LP-ZOL}}\right)\times 100\%$$

W_columns_ is the measured amount of ZOL in the liposome suspension after columns, W_total_ is the measured amount of ZOL in the equal volume of liposome suspensions before columns. W_NGR-PEG-LP-ZOL_ is the measured amount NGR-PEG-LP-ZOL lyophilized powder.

### Antitumor efficacy *in vivo*

This study was approved by the Animal Ethic Committee of Zhejiang University. In detail, MDA-MB-231 cells (1^*^10^6^) resuspended in 10μl PBS were injected intramuscularly into the right hind limb of BALB/c nude (nu/nu) mice. After 14 days (when a tumor mass of about 300 mg was evident), these were divided randomly into seven groups (n=6, each group): (1) Control group (physiological saline, i.v.), (2) ZOL-C (0.1 mg/kg, i.v., q7d), (3) ZOL–M (ZOL equivalent dose of 0.025 mg/kg, i.v., q2d), (4) PEG-LP-ZOL-C (ZOL equivalent dose of 0.1 mg/kg, i.v., q7d), (5) PEG-LP-ZOL-M (ZOL equivalent dose of 0.025 mg/kg, i.v., q2d), (6) NGR-PEG-LP-ZOL-C (ZOL equivalent dose of 0.1 mg/kg, i.v., q7d), (7) NGR-PEG-LP-ZOL-M (ZOL equivalent dose of 0.025 mg/kg, i.v., q2d). The total dose of ZOL in all therapeutic groups was 0.4 mg/kg. All drugs were administered after dissolving in water.

Two dimensional tumor sizes were measured three times a week by using a caliper and tumor weight was calculated using the following formula: a×b^2^/2, where a and b are the long and short diameters of the tumor, respectively. The following end-points were assessed: percent tumor weight inhibition (TWI%) was calculated by,

$$TWI\%=\left[1-\frac{mean\;tumor\;weight\;of\;treated\;mice}{mean\;tumor\;weight\;of\;controls}\right]\times 100\%$$

The harvested tumors were divided into two parts, one of the tumor tissue is fixed in formalin solution and paraffin-embedded for immunohistochemical analysis. The other tumor tissue was dispersed into single tumor cell suspensions, and then analyzed using a Flow Cytometer.

### Histology and immunohistochemistry

Briefly, sections from fixed tumor tissues were cut at a thickness of 3-5μm, mounted on glass and dried overnight at 37°C. All sections were then de-paraffinized in xylene, rehydrated through a graded alcohol series and washed in PBS. This buffer was used for all subsequent washes and for dilution of the antibodies. Tumor tissue sections were stained by immunohistochemistry with anti-CD31 antibody or anti-CD206 antibody.

The MVD was evaluated by Weidner method. Firstly, the tumor sections were scanned at x100 magnification to identify the region of the section with highest microvascular density (so called “hotspot”); this area was then counted at a magnification of ×200 for the microvasculature highlighted by CD31.

### Flow cytometry

Tumors were excised and minced using a scalpel blade and transferred into pre-warmed dissociation buffer [800 μl 1X Collagenas/Hyaluronidase contain 3000 U/mL Collagenase, 1000 U/mL Hyaluronidase, DMEM (1000 mg D-glucose/L)] for 30 min at 37 °C with agitation. The single tumor cell suspensions were stained with F4/80-APC CD206 (1:200 dilution) for 20 min on ice, washed 3 times with PBS, and then analyzed using a Thermo Attune Flow cytometer. The percentage of CD206-positive cells was analyzed using the Flow Jo software.

### Real-time qPCR

After the tumors were excised from breast cancer cell line, MDA-MB-231 tumor-bearing mice, the total RNA was isolated from the tumors using the NucleoSpin RNA L (Macherey-Nagel). RNA yield and purity were checked by spectrometric measurements at 260 and 280 nm. Reverse transcription quantitative polymerase chain reaction (RT-qPCR) amplification was carried out according to the kit protocol. For the amplification of mouse Arg1, the primers, 5’-CTCCAAGCCAAAGTCCTTAGAG-3’, and, 5’-AGGAGCTGTCATTAGGGACTC-3’, were used. For amplification of CCL3, the primers 5’-TTCTCTGTACCATGACACTCTGC-3’, and 5’-CGTGGAATCTTCCGGCTGTAG-3’ were used. For amplification of CCL22, the primers 5’-AGGTCCCTATGGTGCCAATGT-3’, and 5’-CGGCAGGATTTGAGGTCCA-3’ were used. For the amplification of Msr2, the primers 5’-CTTCTGGTCTTCGCTCCTGTC-3’, and 5’-ATGGTGAGCTTGAAGCACTG-3’ were used. For the amplification of FIZZ1, the primers 5’-CCAATCCAGCAGTCATCCCA-3’, and 5’-ACCCAGTAGCAGTCATCCCA-3’ were used. For the amplification of mouse β-actin, the primers β-actin-FW, 5’-TGCTGTCCCTGTATGCCTCT-3’, and β-actin-RW, 5’-TTGATGTCACGCACGATTTC-3’, were used. The qPCR products were analyzed by 1.5% agarose gel electrophoresis in a Tris-Borate-EDTA (TBE) buffer. The products were then visualized by ethidium bromide staining. Real-time PCR was performed on the corresponding cDNA synthesized from each sample described above. The optimized settings were transferred to real-time PCR protocols on iCycler MyiQ detection systems (Bio-Rad CFX Connect™, USA), and SYBR Green I assay (iQ^TM^ SYBER Green Supermix, Bio-Rad Laboratories) was used for quantification. Samples were run in triplicate.

### Statistical analysis and research experience

Data are presented as mean ± standard deviation (SD). SPSS 19.0 was used to determine the significance among groups. P<0.05 was considered to be statistically significant.
